# Factors associated with limited health literacy in a culturally diverse population in Finland: a cross-sectional study

**DOI:** 10.1093/eurpub/ckag130

**Published:** 2026-07-16

**Authors:** Tyler C Prinkey, Annamari Lundqvist, Regina García Velázquez, Hannamaria Kuusio, Robert Griebler, Josefin M Wångdahl, Leena Paakari, Natalia Skogberg

**Affiliations:** Department of Healthcare and Social Welfare, Finnish Institute for Health and Welfare, Helsinki, Finland; Department of Public Health, Finnish Institute for Health and Welfare, Helsinki, Finland; Department of Healthcare and Social Welfare, Finnish Institute for Health and Welfare, Helsinki, Finland; Department of Healthcare and Social Welfare, Finnish Institute for Health and Welfare, Helsinki, Finland; Competence Centre Health Promotion and Healthcare, Austrian National Public Health Institute, Vienna, Austria; Aging Research Center, Karolinska Institutet & Stockholm University, Stockholm, Sweden; Department of Public Health and Caring Sciences, Uppsala University, Uppsala, Sweden; The Faculty of Sport and Health Sciences, University of Jyväskylä, Jyväskylä, Finland; Department of Healthcare and Social Welfare, Finnish Institute for Health and Welfare, Helsinki, Finland

## Abstract

Limited health literacy (HL) has been associated with many adverse health outcomes. Limited HL contributes to adverse outcomes; however, evidence on effective strategies to enhance it remains limited. In this study, we examined HL prevalence in the general Finnish and migrant origin populations and investigate which sociodemographic, health-related, and migration-related factors are associated with limited HL. Data were collected from 2022 to 2023 as part of the cross-sectional population surveys Terve Suomi and MoniSuomi. The analysis included individuals aged 20–74 years (*n* = 9717), with HL assessed using the HLS_19_-Q12. Logistic regressions were used to investigate the association between population, sex, age, living alone, region of residence, education, economic activity, income difficulties, language skills, length of residence, and self-rated health (SRH) with limited HL. A prevalence of limited HL was observed in 15% (95% CI: 13.2–15.9) of the general population and 42% (95% CI: 40.1–43.4) of the migrant-origin population in Finland. Across both groups, limited HL was more likely among individuals with a migration background, unemployment or student status, income difficulties, living alone, lower educational attainment, and average or poor SRH. Among migrants, limited HL was additionally associated with less-than-excellent proficiency in Finnish or Swedish. Limited HL is much more prevalent among the migrant-origin population, highlighting substantial disparities linked to socioeconomic disadvantage, living circumstances, and language proficiency. Earlier efforts to promote HL following migration and greater language inclusion is needed.

## Introduction

Health literacy (HL) is recognized as a social determinant of health [1] and is best understood as a shared responsibility between individuals, local governments, and health systems [2]. HL has been defined as the knowledge, motivation and competencies to access, understand, appraise, and apply health information to make decisions in everyday life concerning healthcare, disease prevention and health promotion to maintain or improve health and quality of life [3]. Limited HL has been shown to be associated with poor health, risk and disease behaviors, higher mortality, and inadequate and increased health care utilization [4–7]. Increasing HL can serve to reduce health inequalities [8] and better manage chronic illnesses [9].

Across European countries, the average prevalence of limited HL has been found to be 63% [10]. In the Nordic region, the prevalence ranges from 51% in Norway to 58% in Denmark [10]. There is strong evidence of a social gradient of HL [11], with a higher socioeconomic status (SES) being associated with a reduced likelihood of limited HL [12, 13], and better access to and application of information [14]. In fact, financial deprivation has been found to be one of the strongest predictors of the social gradient [7]. Some studies have also shown that limited HL is associated with lower educational attainment [12, 13], living alone [15], older age and poor self-rated health (SRH) [12], being male [16], and living in a rural setting compared to an urban setting [17].

Populations in vulnerable situations are distinct subgroups within the broader society that, due to a variety of factors (social, economic, political, structural, geographic, and historical), face heighted exposure to risks which places them at a disadvantage in terms of health and access to healthcare [18]. One subgroup at increased risk of vulnerabilities are persons of migrant-origin. Although research conducted in some EU countries has investigated migrant HL and found associations between limited HL and lower language skills [13, 19], lower educational attainment and economic status [19], and shorter length of residence [20], evidence is limited and, in some cases, inconsistent across studies. This is of particular importance because some research has found persons of migrant origin may be at increased risk for limited HL compared to their respective general population [21]. Furthermore, research on persons of migrant-origin should include a diversity–sensitivity approach that highlights migrant-specific needs and recognizes individual differences [22].

In Finland, research on HL among the Finnish population so far has focused on adolescents [23] and older populations [24]. There is currently no data on the distribution of HL levels and factors associated with these in the working-age adult population. Moreover, no research exists that highlights HL differences between the general population and the migrant-origin population in Finland.

The aim of this research is to examine the distribution of HL levels in the general and migrant-origin populations in Finland, as well as investigate the association of sociodemographic and health-related factors with limited HL. This study’s findings are expected to inform policy recommendations and interventions aimed at reducing health inequities.

## Methods

### Study sample

The current study uses data collected from the cross-sectional MoniSuomi [Diverse Finland] Survey [25] and the Terve Suomi [Healthy Finland] Survey [26]. The MoniSuomi Survey, conducted from September 2022 until March 2023, aimed to provide reliable and up-to-date health-related data on the migrant-origin population in Finland. A random sample of 18 600 people aged 20–74 years, who had lived in Finland for at least 1 year and were born abroad to parents who were also born abroad was drawn from the Digital and Population and Data Services Agency. From the total sample, 7838 persons participated in the study (representing a response rate of 44%).

The MoniSuomi Survey was translated from Finnish into 19 languages [Arabic, Kurdish (Sorani), German, English, Spanish, Estonian, Farsi, Finnish, French, Polish, Dari, Russian, Somali, Albanian, Swedish, Thai, Turkish, Ukrainian, Vietnamese, and Chinese] resulting in 76% of respondents receiving the material in their mother tongue in addition to Finnish or Swedish. Translations were made by a professional translator, proofread, and then checked by a native speaker or professional translator. The data were primarily collected via an online questionnaire (*n* = 4698) and supplemented by a paper questionnaire (*n* = 2649) and telephone interviews (*n* = 491). Telephone interviews were significantly shorter than both online and paper questionnaires and did not include HL questions. The final sample consisted of respondents who answered the HL items and whose HL scores could be calculated (*n* = 5929; response rate of 81%).

The Terve Suomi Survey (2022–23) for which a random sample of the general population (*n* = 61 600, a response rate of 46%) was drawn followed a protocol similar to that of the MoniSuomi Survey. A representative sub-sample of 9973 (participation rate 58%) individuals was subsequently invited to participate in a health examination which also included a questionnaire assessing HL. For the present study, data from the Terve Suomi sub-sample were restricted to individuals aged 20–74 years, to match the age range of MoniSuomi respondents, and whose HL scores could be calculated (*n* = 3788).

All participants gave informed consent in their native language (whenever possible, otherwise in Finnish, Swedish, or English) to take part in either the MoniSuomi (THL/1270/6.02.01/2022) or the Terve Suomi study (THL/72/6.02.01/2022) which received ethical permissions from Finnish Institute for Health and Welfare′s (THL) Ethics Committee. The Terve Suomi study obtained additional review by the Helsinki and Uusimaa Hospital District Regional Committee on Medical Research Ethics for the health examination portion of the study (decision number HUS/900/2022).

### Variables

HL was measured using the 12-item short form questionnaire of the HL Survey (HLS_19_-Q12) for measuring general HL [10], which consists of 12 items reflecting three domains of HL (health care, disease prevention, health promotion). Items were rated on a four-point Likert scale regarding the experienced difficulty of the 12 HL tasks (1 = very easy, 2 = fairly easy, 3 = fairly difficult, 4 = very difficult; complemented by a “don’t know” category) related to accessing, understanding, appraising and applying health information [27]. P-type scores for general HL were calculated and used in the formation of “HL categories” following the protocol as outlined in The HLS_19_ Consortium of the WHO Action Network M-POHL factsheet [28]:

- *Excellent*: > 83.33
- *Sufficient*: > 66.67 and ≤ 83.33
- *Problematic*: > 50 and ≤ 66.67
- *Inadequate*: ≤ 50

“HL categories” were transformed into a binary HL variable highlighting excellent/sufficient HL versus problematic/inadequate HL. The latter category is also referred to as limited HL [10].

Information on “age” (grouped to 20–34, 35–49, and 50+ years; based on the distribution of our sample), “sex,” and “length of residence in Finland” (1–4, 5–10, and 11+ years) was obtained from the Finnish Population Register. A binary variable “population” (General vs Migrant-origin; based on which survey participant responded to) was created to highlight population differences. Participants from the MoniSuomi survey were further grouped into five regions of origin (i) Russia or the Former Soviet Union (FSU); (ii) Estonia; (iii) The Rest of Europe, North America and Oceania; (iv) The Middle East and Africa; and (v) Asia and Latin America) adapted from the United Nations area code grouping [29].

Self-reported data included information on “education” (comprehensive school or less, secondary education, higher education), “self-rated health” (SRH) (good/fairly good, average/poor), “living alone” (yes, no), “Finnish/Swedish language skills” (intermediate or less versus excellent language skills), and a binary self-reported “income difficulties” variable comprised of answers related to not having enough money for food, medicine, and doctor visits. A self-reported “economic activity” variable (economically active/currently working, student, “other”) was created where “other” refers to those who are retired on an old age pension, receive disability pension, are unemployed/laid off, are on family leave, or are a stay-at-home parent. Another self-reported “region of residence” variable was formed based on the Finnish Environment Institute’s Urban-rural classification [30] which was simplified into three categories (urban, highly populated municipality, rural) based on the degree of urbanization.

### Statistical analyses

**Figure 1.**
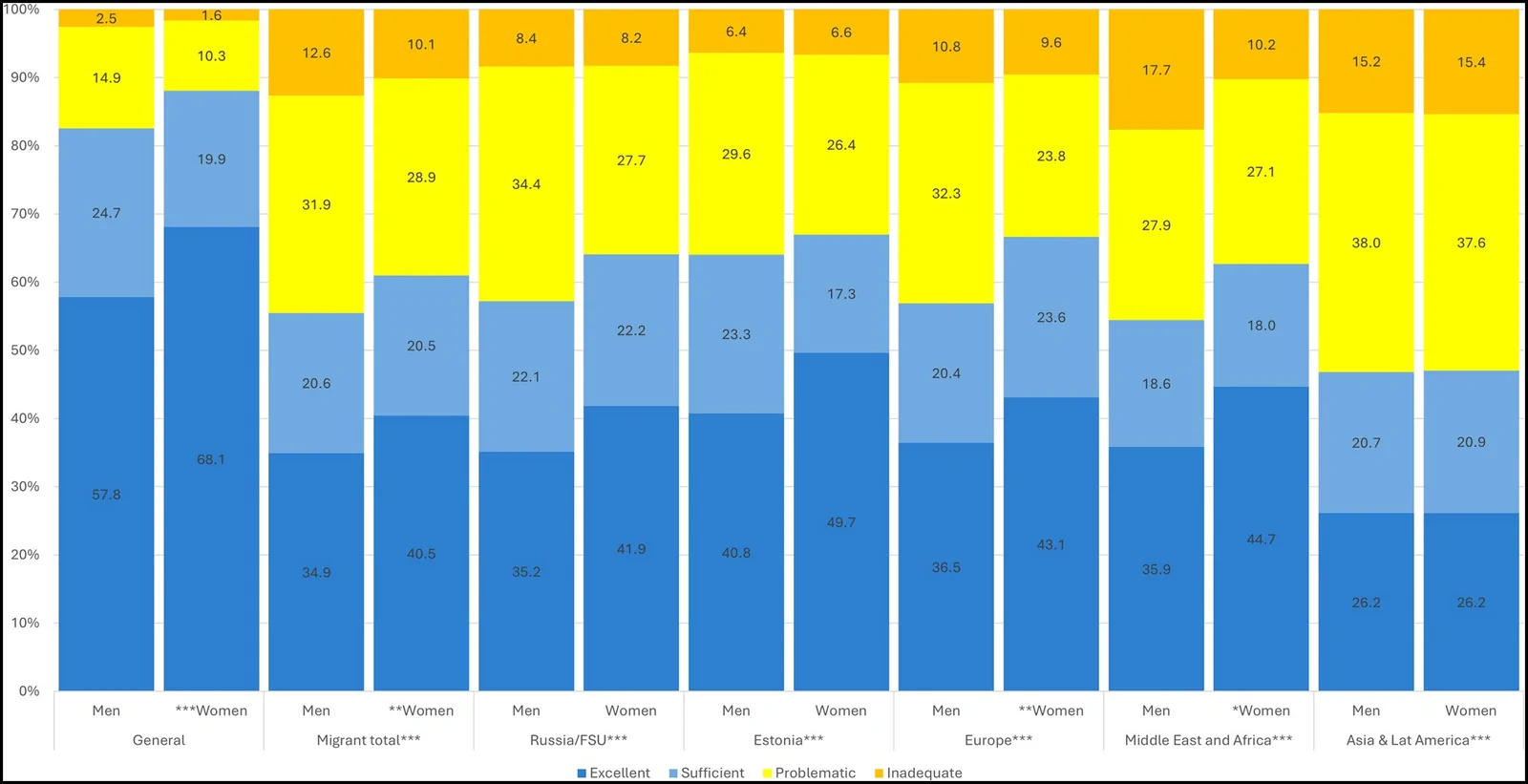
Distribution of HL levels across sex and population/migrant groups. Row wise proportion calculations were used to visualize differences in HL level within each group. Design adjusted Rao & Scott Chi square tests were conducted to assess statistically significant variations in limited HL by sex and between the general population and migrant population/groups. *P < .05; **P < .01; **P < .001.

The distribution of HL levels can be seen in Table 1 as well as in Fig. 1. In Fig. 1, row-wise proportion calculations were carried out to visualize differences in HL level across sex and population/migrant groups. Design-adjusted Rao & Scott Chi-square tests were conducted to assess statistically significant variations in limited HL by sex and between the general population and migrant population/groups.

**Table 1.** Descriptive statistics of study participants

	General population	Migrant-origin total	Russia or FSU	Estonia	Rest of Europe	Middle East and Africa	Asia and Latin America
	*n* = 3788	*n* = 5929	*n* = 1334	*n* = 608	*n* = 1314	*n* = 1190	*n* = 1483
	*n*(%)	*n*(%)	*n*(%)	*n*(%)	*n*(%)	*n*(%)	*n*(%)
Men	1628 (47.9)	2729 (50.2)	472 (39.6)	237 (45.1)	736 (58.0)	710 (62.2)	574 (42.9)
Age							
20–34	720 (25.5)	1770 (31.4)	265 (24.3)	116 (21.9)	392 (30.8)	451 (39.0)	546 (37.7)
35–49	950 (26.7)	2475 (42.4)	479 (39.8)	227 (38.2)	559 (45.5)	522 (41.7)	688 (46.7)
50+	2118 (47.8)	1684 (26.1)	590 (35.9)	265 (39.9)	363 (23.7)	217 (19.2)	249 (15.7)
Education							
Higher education	1810 (63.0)	3106 (44.5)	743 (54.6)	152 (18.4)	770 (52.3)	518 (34.7)	923 (58.1)
Secondary education	749 (30.4)	1918 (36.2)	482 (36.5)	287 (48.1)	424 (37.8)	406 (37.6)	319 (23.3)
Comprehensive school or less	176 (6.5)	835 (19.2)	107 (9.0)	162 (33.5)	105 (9.9)	245 (27.7)	216 (18.5)
Economic activity							
Economically active	1592 (58.1)	3776 (65.7)	780 (62.7)	443 (76.4)	935 (72.4)	617 (52.2)	1001 (72.3)
Student	171 (8.8)	616 (10.7)	102 (8.6)	22 (4.1)	81 (6.6)	234 (19.5)	177 (10.5)
Other^a^	975 (33.0)	1382 (23.6)	421 (28.7)	130 (19.5)	279 (21.0)	301 (28.3)	251 (17.2)
Income difficulties	359 (17.3)	1831 (33.6)	431 (33.7)	177 (27.8)	295 (24.0)	528 (46.6)	400 (30.1)
Live alone	623 (27.2)	1163 (22.7)	287 (24.7)	158 (26.8)	186 (16.4)	285 (26.9)	247 (18.1)
Region of residence							
City	2366 (64.3)	5065 (85.1)	1091(82.4)	473 (76.0)	1074 (81.5)	1105 (93.0)	1322 (88.5)
Highly populated municipality	740 (19.5)	457 (7.2)	120 (7.1)	85 (14.9)	138 (8.8)	43 (3.5)	71 (4.7)
Rural	682 (16.2)	407 (7.7)	123 (10.6)	50 (9.2)	102 (9.6)	42 (3.5)	90 (6.8)
Poor/average SRH	1066 (31.9)	1842 (31.1)	498 (33.4)	226 (37.1)	345 (26.7)	334 (29.0)	439 (30.9)
Length of residence							
1–4	NA	1444 (14.9)	198 (7.9)	59 (7.0)	355 (18.9)	335 (19.5)	497 (19.3)
5–10	NA	1781 (34.6)	308 (22.5)	212 (39.8)	368 (33.9)	429 (39.5)	464 (38.9)
11+	NA	2704 (50.5)	828 (69.5)	337 (53.3)	591 (47.2)	426 (41.0)	522 (41.9)
Intermediate language skills or less	NA	4282 (68.0)	853 (55.7)	291 (47.9)	917 (69.9)	917 (75.1)	1304 (87.0)
Good HL^b^	3289 (85.4)	3382 (58.2)	754 (61.4)	405 (65.7)	809 (61.0)	700 (57.6)	714 (47.0)
Excellent	2488 (63.2)	2101 (37.7)	448 (39.2)	270 (45.6)	500 (39.3)	471 (39.2)	412 (26.2)
Sufficient	801 (22.2)	1281 (20.6)	306 (22.2)	135 (20.0)	309 (21.8)	229 (18.3)	302 (20.8)
Limited HL^c^	499 (14.6)	2547 (41.8)	580 (38.6)	203 (34.3)	505 (39.0)	490 (42.4)	769 (53.0)
Problematic	440 (12.5)	1889 (30.4)	455 (30.3)	169 (27.8)	380 (28.7)	330 (27.6)	555 (37.8)
Inadequate	59 (2.0)	658 (11.4)	125 (8.3)	34 (6.5)	125 (10.3)	160 (14.8)	214 (15.3)

a “Other” refers to those who are retired on an old age pension, receive disability, are unemployed/laid off, are on family leave, or are a stay-at-home parent.

b Good HL is “Excellent” and “Sufficient” HL categories combined.

c Limited HL is “Problematic” and “Inadequate” HL categories combined.

Multiple logistic regressions, with a nested structure, were run to investigate associations between included variables and limited HL in Table 2. As an aim of this study was to examine the distribution of HL levels in the general and migrant-origin populations in Finland, rather than for individual migrant-origin groups, regression analyses were conducted at the population level. Interaction testing for sex, age, and population was conducted across all models. After building the final models with all initially significant interaction terms, we re-evaluated each interaction to assess its contribution in the context of the full model. Interaction terms that were no longer significant were excluded to improve parsimony and model stability. Differences in population sample sizes were accounted for through the inclusion of interaction terms between population and living alone and education.

**Table 2.** Multiple logistic regression analysis of factors associated with limited HL

	Model 1^a^ OR (95 % CI)	Model 2^b^ OR (95 % CI)	Model 3^c^ OR (95 % CI)	Model 4^d^ OR (95 % CI)
Population				
General population	1.00	1.00	1.00	1.00
Migrant-origin population	5.54 (4.59, 6.67)***	6.22 (4.87, 7.94)***	6.43 (5.03, 8.23)***	2.77 (2.09, 3.68)***
Sex				
Men	1.00	1.00	1.00	1.00
Women	0.78 (0.69, 0.89)***	0.74 (0.64, 0.84)***	0.80 (0.67, 0.94)**	0.88 (0.74, 1.05)
Age				
50+	1.00		1.00	1.00
35–49	0.97 (0.84, 1.13)	1.05 (0.89, 1.24)	1.17 (0.99, 1.38)	1.05 (0.88, 1.26)
20–34	0.87 (0.74, 1.02)	0.94 (0.79, 1.13)	1.09 (0.91, 1.32)	1.04 (0.84, 1.28)
Living alone				
No	1.00	1.00	1.00	1.00
Yes	2.03 (1.52, 2.70)***	1.76 (1.30, 2.39)***	1.62 (1.19, 2.21)**	1.63 (1.20, 2.23)**
Education				
Higher education	1.00	1.00	1.00	1.00
Secondary education	NA	1.45 (1.06, 1.97)*	1.41 (1.03, 1.93)*	1.40 (1.02, 1.92)*
Basic education or less	NA	1.43 (0.86, 2.37)	1.44 (0.86, 2.40)	1.41 (0.85, 2.36)
Economic activity				
Economically active	1.00	1.00	1.00	1.00
Student	NA	0.82 (0.64, 1.04)	0.81 (0.63, 1.04)	0.73 (0.57, 0.94)**
Other^e^	NA	1.47 (1.25, 1.72)***	1.33 (1.13, 1.57)***	1.27 (1.07, 1.51)**
Region of residence				
Urban	1.00	1.00	1.00	1.00
Highly populated municipality	NA	0.86 (0.68, 1.10)	0.87 (0.68, 1.11)	0.95 (0.74, 1.23)
Rural	NA	1.01 (0.77, 1.33)	0.99 (0.75, 1.31)	1.02 (0.76, 1.38)
Income difficulties				
No	1.00	1.00	1.00	1.00
Yes	NA	1.93 (1.67, 2.23)***	1.67 (1.44, 1.95)***	1.68 (1.44, 1.96)***
SRH				
Good/fairly good	1.00	1.00	1.00	1.00
Average or worse	NA	NA	2.56 (2.07, 3.16)***	2.49 (2.01, 3.10)***
Interaction Population and Living alone				
Migrant × Living alone	0.55 (0.39, 0.77)***	0.58 (0.41, 0.82)**	0.63 (0.44, 0.90)**	0.62 (0.43, 0.89)**
Interaction Population and Education				
Migrant × Basic education or less	NA	0.92 (0.53, 1.58)	0.91 (0.52, 1.59)	0.82 (0.47, 1.44)
Migrant × Secondary education	NA	0.52 (0.37, 0.74)***	0.51 (0.36, 0.73)***	0.56 (0.39, 0.79)***
Interaction Sex and SRH				
Women × Average or worse SRH	NA	NA	0.73 (0.55, 0.96)*	0.71 (0.53, 0.95)*
	**MIGRATION-RELATED FACTORS**			
Length of residence in Finland				
11+ years	1.00	1.00	1.00	1.00
5–10 years	NA	NA	NA	1.20 (0.99, 1.45)
1–4 years	NA	NA	NA	1.23 (0.98, 1.55)
Language skills				
Excellent	1.00	1.00	1.00	1.00
Intermediate or less	NA	NA	NA	2.92 (2.40, 3.55)***
Nagelkerke *R* squared	0.12	0.16	0.19	0.24
No observations	8239	7821	7786	7767

a Adjusted for population, age, sex, and living alone. Interaction term population × living alone was included in this model.

b Adjusted for population, age, sex, living alone, education, economic activity, region of residence, and income difficulties. Interaction terms population × living alone, and population × education were included in this model.

c Adjusted for population, age, sex, living alone, education, economic activity, region of residence, income difficulties, and SRH. Interaction terms population × living alone, population × education, and sex × SRH were included in this model.

d Adjusted for population, age, sex, living alone, education, economic activity, region of residence, income difficulties, SRH, length of residence, and language skills. Interaction terms population × living alone, population × education, and sex × SRH were included in this model.

e “Other” refers to those who are retired on an old age pension, receive disability, are unemployed/laid off, are on family leave, or are a stay-at-home parent.

* *P* value < .05;

** *P* value < .01;

*** *P* value < .001.

To avoid excluding general population cases from the analysis in Model 4, we interacted migrant-specific predictors with the population group variable. This approach allowed the model to estimate the effects of these variables only within the migrant population, while retaining general population respondents in the full model. This strategy maintains sample size and respects the conceptual relevance of these variables.

Ultimately, Model 1 (adjusting for population, sex, age and living alone) included an interaction term between population and living alone, Model 2 (adjusting for population, sex, age, living alone, education, economic activity, and income difficulties) included interaction terms between population and living alone, and population and education, and Models 3 (adjusting for all variables in Model 2 and adding SRH) and 4 (adjusting for all variables in Model 3 and adding migration-related factors language skills and length of residence) included interaction terms between population and living alone, population and education, and sex and SRH. Model comparison using design-based Rao-Scott adjusted Likelihood Ratio Tests (LRT) indicated that including interaction terms improved model fit for all models (*P* < .001).

Odds ratios (ORs) and their corresponding 95% confidence intervals (CIs) were calculated. *P* value were calculated using the Wald test statistic. Non-response bias was reduced by using inverse probability weights, which also took varying sampling probabilities into account [31]. All statistical analyses were performed using the R “survey“ package [32]. The significance level was set at *α* = 0.05.

## Results

Descriptive characteristics of study participants are presented in Table 1. The general population had a higher percentage of persons aged 50+ than the migrant-origin population (48% and 26%, respectively), and more persons in the general population were other than economically active or students compared with the migrant-origin population (33% and 24%, respectively). The general population had a higher prevalence of persons with higher education (63%) and experienced less income difficulties (17%) compared to the migrant-origin population (45% and 34%, respectively). Most participants lived in a city (64% of the general population, 85% of the migrant-origin population).

### Distribution of HL level by origin and sex

Prevalence of limited HL was 15% in the general population and 42% in the migrant origin population. Among the migrant groups, prevalence varied greatly. Limited HL ranged from 34% in the Estonian migrant group to 53% in the Asia and Latin America group (see Table 1). Significant differences in limited HL were found between general and migrant-origin populations (*P* < .001) as well as between every migrant group and the general population (*P* < .001). Moreover, a noticeable difference can be seen in extremes of the HL categorizations between the general- and migrant-origin populations. For the “Excellent” categorization, the general population had 63% whereas this proportion was 38% for the migrant-origin population. The “Inadequate” HL categorization corresponded to only 2% for the general population and 11% for the migrant-origin population.

Differences in HL level by origin and sex are presented in Fig. 1. Design-adjusted Rao & Scott Chi-square tests found significant sex differences in limited HL among the general population (17% and 12% for men and women, respectively), and the migrant-origin population (45% and 39% for men and women, respectively). Sex differences were especially pronounced in the European migrant group (43% and 33% for men and women, respectively) and the Middle East and Africa migrant group (46% and 37% for men and women, respectively).

### Factors associated with limited HL

Results were consistent across the first three regression models, finding an association between being of migrant origin and living alone with increased odds of having limited HL. Being female was associated with decreased odds of having limited HL. In models two and three, having secondary education compared to higher education, being other than economically active or a student, and experiencing income difficulties was associated with increased odds of having limited HL.

Model 4, a full model which further included migration-related factors (i.e. language skills and length of residence), increased the explained variance from previous models to a Nagelkerke R2 of 0.24 suggesting that migration-related characteristics contributed additional explanatory value beyond sociodemographic, socioeconomic and health-related factors. In this model, being of migrant-origin (OR= 2.77, 95% CI = 2.09–3.68), reporting average or worse SRH (OR = 2.49, 95% CI = 2.01–3.10), having experienced income difficulties (OR = 1.68, 95% CI = 1.44–1.96) living alone (OR = 1.63, 95% CI = 1.20–2.2), having secondary compared to higher education (OR = 1.40, 95% CI = 1.02–1.92), and belonging to the economic activity category “other” compared to those who are economically active (OR = 1.27, 95% CI = 1.07–1.51) increased the odds of having limited HL. Being a student compared to economically active (OR = 0.73, 95% CI = 0.57–0.94), was associated with reduced odds of having limited HL. This finding differs from all previous models which did not find a significant difference.

Migrants with intermediate or less language skills had almost three times greater odds of having limited HL (OR = 2.92, 95% CI = 2.40–3.55) compared to those with excellent language skills. Although being female was associated with lower odds of limited HL in Model 3 (OR = 0.80, 95% CI = 0.67–0.94), it lost significance in Model 4 (OR = 0.88, 95% CI = 0.74–1.05) with the inclusion of migration-related factors. Length of residence was not significantly associated with lower odds of limited HL in this study.

All models tested the interaction between population and living alone and found a reduced odd of having limited HL among persons of migrant origin living alone compared to those in the general population (see Table 2). Models 2 through 4 found a reduced odd of having limited HL among migrants with secondary education compared to persons in the general population with secondary education. Models 3 and 4 found a reduced odd of having limited HL among women with average or worse SRH compared to men with average or worse SRH.

## Discussion

Our study aimed to examine the prevalence of limited HL in Finland and to evaluate which sociodemographic, health-related, and migration-related factors are associated with limited HL. The prevalence of limited HL among the general population in Finland was found to be low (15%) whereas the migrant-origin population′s prevalence of limited HL (42%) in this study aligned more closely with previous research, despite also being lower. Our study supports previous research suggesting migrant-origin populations may have increased odds of having limited HL [19, 21] as our study found being of migrant-origin was associated with three times greater odds of having limited HL compared to the general population. Moreover, all migrant groups in this study were found to have a significantly higher prevalence of limited HL compared to the general population.

Although findings from studies among persons of migrant-origin found shorter length of residence [20] to be associated with higher odds of limited HL, this association was not found in the present study. Other research which found lower language skills [13, 19] to be associated with higher odds of limited HL were supported by our study. In our study, 68% of the migrant population had only intermediate or lower Finnish/Swedish language skills, which is representative of the Finnish migrant-origin population [33]. Backed by our analyses, and previous research finding an association between language skills and adequate access to information [34], language was also found to be a very strong predictor of limited HL in migrant-origin persons in Finland.

Previous research on migrants found that HL could be attenuated by field of work, with working in the healthcare sector being a protective factor against having limited HL [35]. As of 2024, 16% of employed persons with foreign background in Finland were found to work in the field of human health and social work [36] which could help explain the prevalence of HL in the Finnish migrant-origin population as well.

Our study also found higher odds of limited HL among those who live alone, those who are not students or currently economically active, those who have experienced income difficulties, and those with average or worse SRH. These results support previous studies that found higher odds of limited HL among persons with lower socioeconomic status [12, 13], poorer SRH [12], and who live alone [15]. Although some previous research has found being female to be associated with reduced odds of having limited HL [16], our study’s results do not support this.

Interestingly, with the inclusion of the interaction term population x living alone, it was discovered that persons with a migrant-background who live alone had lower odds of having limited HL than persons in the general population who live alone. This suggests that some aspects of social support may work differently between these populations. Similarly, women with average or worse SRH had lower odds than men with average or worse SRH of having limited HL. Although these interactions had borderline significance levels, they could be of interest for further research.

The low prevalence of limited HL found in this study may also be partially explained by high educational attainment. Educational attainment in Finland is high with around 75% of those aged 15 or over have completed post-comprehensive level qualifications [37]. Our study population had a very high prevalence of persons with at least secondary education in the general population (93%) and in the migrant-origin population (81%). As lower education attainment is associated with limited HL [12, 13], our highly educated population would be more likely to have higher HL on average.

Although some studies have found older age to be associated with limited HL [12], the oldest age group in our study, 50 years or older, comprised mainly person between the ages of 50–65, due to few cases of persons in our sample being between the ages of 66–74. This could translate to higher HL scores as our combined population skews younger and may also explain why there were no significant differences in odds of limited HL between age groups.

### Strengths and limitations

A major strength of this study is having data from two nationally representative surveys using a highly language-inclusive design. The low prevalence of limited HL found in this study, however, may have been influenced by using a newly translated versions of the English-language HLS_19_-Q12 which included middle rating options that had the word “fairly” in them and included a “don’t know” response category. The additional response category “don’t know” may have inadvertently led to a higher prevalence of the inability to calculate scores, as was found in a 2014 study in Sweden [38] and may have introduced a bias towards higher levels of HL.

An additional factor to consider is that the response order of the HLS19-Q12 differed between the surveys: Terve Suomi ranged from “very difficult” to “very easy,” whereas MoniSuomi used the reverse order. With these points in mind, previous research in Finland on adolescents found statistically significantly higher mean HL scores than many other countries in Europe [23] and a prevalence of high HL among older adults of 64% [24] suggesting that HL may be higher on average in Finland.

Another limitation to consider is limited sample size in some subgroups, which may have led to reduced statistical power, particularly for interaction analyses. Moreover, our regional groupings were constrained by data availability, as several migrant-origin subgroups were too small to allow separate analyses or reliable reporting. Consequently, some migrant-origin groups are broad and internally heterogeneous, which limits the interpretability of comparisons across groups. The grouping strategy, therefore, reflects a pragmatic balance between analytic feasibility and the ability to examine broader patterns in migrant-origin differences. Additionally, the use of self-reported measures may have introduced reporting bias. Finally, although data are weighted to be nationally representative, findings may not be fully generalizable to all migrant populations or settings.

## Conclusion

Health systems should be designed to make understanding, accessing, and acting on health information easier by reducing overall complexity and through providing a supportive environment. Earlier and more targeted efforts to include migrant-origin persons should be made following migration to Finland. Although language courses are a promising avenue for implementing change among those who have migrated to Finland, language interventions alone are not enough. Research in Sweden has discovered that providing additional health communication during civic orientation courses among migrants can increase HL [39]. Other research suggests that HL promotion among migrants should include informal opportunities for health information exchange as well as diverse and multilingual ways to engage with health information [40]. Targeted strategies, such as promoting culturally tailored health information, using more inclusive language in healthcare settings, and addressing socioeconomic barriers, can contribute to improved HL in the Finnish population and, subsequently, better health outcomes.

Conflict of interest: No conflicts of interest to report.

## Data Availability

The data underlying this article cannot be shared publicly due to ethical and legal restrictions (GDPR/privacy of participants). The data will be shared on reasonable request to the corresponding author under a collaboration agreement. Key points Although general HL in Finland is comparatively high in the Finnish-born population, work should be done to address differences in HL across diverse population groups. Systematic change, which is needed to be more inclusive of the factual population diversity in Finland, should consider persons with low SRH, those who experience income difficulties, those who live alone, those not currently working, and persons with low to intermediate Finnish/Swedish language skills. The migrant-origin population living in Finland, could benefit from greater language inclusion and earlier HL promotion efforts following migration.
